# Evidence-Based Practice for Comprehensive Management of Pemphigus Skin Lesions: An Evidence Synthesis Review

**DOI:** 10.3390/jcm15051965

**Published:** 2026-03-04

**Authors:** Lingjie Gao, Xinyue Zhang, Hongwei Yan, Shiyao Dong, Xiaobo Li

**Affiliations:** 1 Department of Dermatology, The First Affiliated Hospital of China Medical University, Shenyang 110001, China; gbecky0719@163.com (L.G.);; 2 Nursing Department, The First Affiliated Hospital of China Medical University, Shenyang 110001, China; 15698829877@163.com (X.Z.);; 3 Department of Urology, The First Affiliated Hospital of China Medical University, Shenyang 110001, China

**Keywords:** pemphigus, skin lesions, management, evidence-based nursing, evidence synthesis

## Abstract

**Objectives**: To identify, evaluate, and synthesize best evidence-based practices for the comprehensive care and management of cutaneous lesions in Pemphigus patients, encompassing assessment, prevention, topical care, health education, and follow-up. This aims to provide evidence-based guidance for clinical practice. **Methods**: Guided by the “6S” evidence model, a systematic search was performed across multiple databases, guideline repositories, and professional organization websites. The literature published from the inception of each database up to 25 February 2025 was considered. Two researchers with training in evidence-based methods independently assessed the quality of included literature, extracted data, and synthesized the evidence. **Results**: A total of 14 publications were included, consisting of 1 clinical decision tool, 6 guidelines, 6 expert consensus documents, and 1 systematic review. From these, 24 evidence recommendations were summarized, organized into five key areas: management principles, skin assessment, lesion care, health education, and recurrence and follow-up. **Conclusions**: This review integrates current best evidence on skin lesion management in Pemphigus into a structured set of recommendations. The findings offer practical, evidence-based guidance for clinical practice and can support the development of standardized care protocols to improve patient outcomes.

## 1. Introduction

Pemphigus is a rare and serious autoimmune blistering disease that affects the skin and mucous membranes. It is characterized by the formation of blisters and erosions. Pemphigus includes Pemphigus vulgaris, Pemphigus foliaceus, paraneoplastic Pemphigus, and IgA Pemphigus [1]. The two most common forms are Pemphigus vulgaris (PV) and Pemphigus foliaceus (PF) [2]. Pemphigus is a chronic condition that leads to considerable skin damage, reduces quality of life, and is associated with high mortality [3].

The management of Pemphigus focuses on suppressing pathogenic antibody production, controlling disease activity to prevent new lesion formation, and promoting the healing of existing blistering skin lesions [4]. Systemic therapies, including corticosteroids, immunosuppressants, and rituximab, constitute the mainstay of treatment [5]. Although these systemic interventions are essential for modulating the underlying autoimmune process, they do not directly facilitate the repair of already established cutaneous and mucosal lesions. Moreover, the immunosuppressive effects of such treatments may elevate the risk of secondary skin infections, which can further impede wound healing. Therefore, the management of skin lesion repair in this chronic disease, along with routine skin care, poses considerable challenges in clinical practice. In addition to systemic therapy, the standardized local management of symptoms—such as blisters, erosions, and cutaneous infections—represents a vital and integral component of comprehensive patient care.

Epidemiological studies report an incidence of 0.42–1.6 per 100,000 people, with a prevalence between 7% and 26% [6,7]. Without treatment, mortality rates can be as high as 90% [8]. Even with systemic therapy, the disease often follows a long-term and relapsing course [7]. Widespread skin lesions impair the barrier function of the skin, increase the risk of complications such as local infection and sepsis, and severely affect daily living.

In clinical practice, although early diagnosis and medical treatment can help control disease progression, the healing of skin damage, prevention of complications, and long-term management rely heavily on nursing care. Important challenges in nursing include non-standard wound care practices that slow healing, a lack of patient self-care knowledge, and inadequate monitoring of treatment-related side effects. These factors often contribute to disease recurrence. At present, relevant nursing recommendations are scattered throughout clinical guidelines and expert consensus documents. There is a lack of integrated, systematic evidence specifically addressing nursing practice. This has hindered the development of a standardized, evidence-based nursing protocol.

This study aims to systematically synthesize evidence on holistic care management of Pemphigus skin lesions, transcending the limitations of drug therapy alone. It encompasses principles of skin management, assessment tools, wound care interventions, patient health education, and long-term monitoring and follow-up. This research provides a scientific basis for clinicians to develop standardized care and management protocols, effectively enhancing the quality of skin lesion management. Ultimately, it seeks to reduce complications while improving patients’ quality of life.

## 2. Materials and Methods

### 2.1. Establish Evidence-Based Questions

The evidence-based question was structured using the PIPOST framework from the JBI Center for Evidence-Based Nursing at Fudan University in Shanghai, China [9]. The model comprised the following elements: P (Population) referred to patients with Pemphigus; I (Intervention) included the assessment, prevention, and management of skin lesions; P (Professionals) involved clinical staff responsible for skin lesion care; O (Outcome) focused on skin lesion improvement and quality of life; S (Setting) encompassed hospitals, rehabilitation centers, and home environments; and T (Type of Evidence) included guidelines, expert consensuses, clinical decision aids, evidence summaries, and systematic reviews. The study was registered with the Evidence-Based Nursing Center of Fudan University (Registration Number: ES20258002).

### 2.2. Evidence Retrieval

Following the “6S” pyramid model for evidence retrieval [10], a systematic top-down search was conducted across multiple sources. These included computerized decision support systems such as BMJ Best Practice and Up to Date; guideline websites including NGC, GIN, SIGN, NICE, China Medical Pulse, and JBI; professional association websites from ASA, European Wound Management Association, and dermatology branches of Chinese medical associations; and databases comprising PubMed, Embase, Cochrane Library, Web of Science, CINAHL, CBM, CNKI, Wan fang, and VIP. The search covered all the literature from database inception through 25 February 2025.

The Chinese search terms we retrieved were “pemphigus/pemphigus vulgaris/pemphigus foliaceus,” “skin damage/lesions/blisters” and “prevention/management/nursing”. The English search terms were “Pemphigus/Pemphigus Vulgaris/Pemphigus Foliaceus/Foliaceus, Pemphigus,” “skin damage/skin injury/Blisters/Bleb/Bulla/Vesication,” and “Prevention/management/nursing”.

The Chinese search strategy using CNKI as an example is as follows: (Subject: Pemphigus + Pemphigus Vulgaris + Pemphigus Foliaceus) AND (Abstract Keywords: Skin Damage + Skin Lesion + Blisters) AND (Abstract Keywords: Prevention + Management + Nursing).

The English search strategy using PubMed is as follows: (((“Pemphigus”[Mesh]) OR (((Pemphigus Vulgaris[Title/Abstract]) OR (Pemphigus Foliaceus[Title/Abstract])) OR (Pemphigus foliaceus[Title/Abstract]))) AND ((skin damage[Title/Abstract]) OR (skin injury[Title/Abstract]))) OR (“Blister” [Mesh])) OR (((Bleb[Title/Abstract]) OR (Vesication[Title/Abstract])) OR (Bulla[Title/Abstract]))) AND (((prevention[Title/Abstract]) OR (management[Title/Abstract])) OR (nursing[Title/Abstract])). Adjust the search formula as needed according to each database’s requirements.

### 2.3. Inclusion and Exclusion Criteria for Literature

Inclusion Criteria: (1) patients diagnosed with Pemphigus of any subtype; (2) studies focusing on assessment, prevention measures, local management, health education, or management of skin or mucosal lesions; (3) study type including guideline, expert consensus, clinical decision aid, evidence summary, or systematic review; and (4) the article was published in Chinese or English.

Exclusion Criteria: (1) Studies discussing only drug therapies without addressing skin care measures; (2) the literature where full-text access was unavailable or literature consisting solely of guideline interpretations; and (3) the literature with low-quality evaluations.

### 2.4. Literature Quality Assessment Criteria and Process

Select the appropriate literature quality assessment tools based on document type. (1) Clinical guidelines are evaluated using the Appraisal of Guidelines for Research and Evaluation II (AGREE II) tool (2017 Edition) [11]. (2) Systematic reviews are assessed using the JBI Center for Evidence-Based Healthcare Systematic Review Tool (2016 Edition) [9]. (3) Expert consensus statements are evaluated using the JBI Expert Consensus Statement Assessment Criteria (2016 version) [12]. (4) Clinical decisions fall under the topic evidence summaries category within the “6S” pyramid. Their quality is assessed using the Critical Appraisal for Summaries of Evidence (CASE) checklist [13]. The literature quality evaluation strictly adheres to established standards. In cases of disagreement, members of the evidence-based practice team collectively discuss and analyze the findings to reach a final consensus.

### 2.5. Evidence Extraction and Synthesis

Two researchers trained in evidence-based methods conducted full-text reviews of the included literature. They independently evaluated study quality and extracted relevant evidence. Guidelines were assessed by four researchers, while other publication types were evaluated by two. To ensure clinical relevance and scientific soundness, nursing experts reviewed and adjusted the evidence based on feasibility, appropriateness, clinical significance, and effectiveness, ensuring standardization and accuracy throughout the evidence grading and integration process.

Evidence integration followed three principles: (1) When evidence conclusions conflicted, higher-level evidence with superior quality and more recent publication dates took precedence. For example, “Recommend reducing pungent foods like garlic, onions, and leeks, avoiding alcohol and hard foods” versus “Maintain a balanced diet; dietary restrictions are not recommended”—the former from an expert consensus [14] and the latter from a newer guideline [15]—prioritizes the latter. (2) When evidence content is complementary, merge it based on logical relationships, such as “Recommend regular follow-up” [16], “Recommend follow-up every 1–2 weeks until disease control, then every 3 months” [17], and “Recommend follow-up every 2–4 weeks, every 4–8 weeks after disease control, and extend to every 8–16 weeks after stabilization” [18]. These three recommendations are combined to form a comprehensive follow-up frequency recommendation; (3) when evidence was consistent, the clearest and most professionally appropriate formulation was selected.

After reviewing the included literature, evidence was classified using the Australian JBI Centre for Evidence-Based Health Care Evidence Grading System (2016) [19]. Evidence was categorized into Levels 1–5, with Level 1 representing the highest quality. If a study already provided evidence grading consistent with this system, it was directly adopted. For studies without grading or using different systems, evidence levels were determined based on the original study design, with priority given to high-quality, recently published evidence from peer-reviewed journals.

## 3. Results

### 3.1. Literature Search Results and General Characteristics of Included Studies

**Figure 1:**
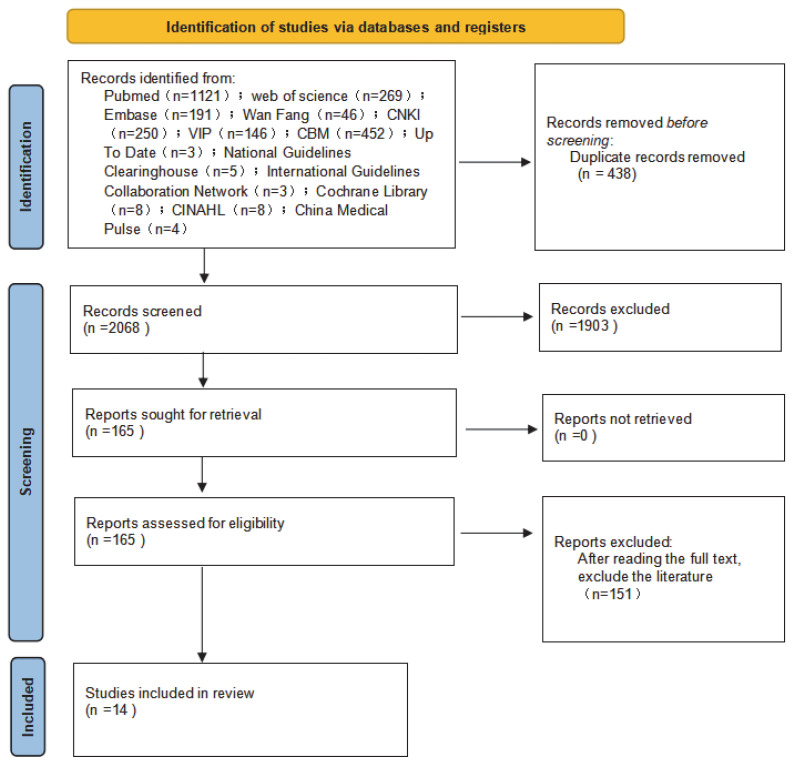
Literature screening process flowchart.

The initial literature search yielded 2506 studies. After excluding duplicates and studies that did not meet the criteria, 14 studies were ultimately included. These comprised: one clinical decision aid [20], six guidelines [15,16,18,21,22,23], six expert consensus documents [3,14,17,24,25,26], and one systematic review [27]. The literature screening flowchart is shown in Figure 1, and the general characteristics of the included studies are summarized in Table 1.

### 3.2. Quality Assessment Results of Included Studies

#### 3.2.1. Quality Assessment Results for Clinical Decision-Making

This study included one clinical decision-making guideline [20], with all items assessed as “Yes” meeting inclusion criteria.

#### 3.2.2. Quality Assessment Results for Guidelines

This study included six guidelines [15,16,18,21,22,23]. The methodological quality assessment results for the guidelines are shown in Table 2. The overall quality assessment was rated as Grade B, and all were included.

#### 3.2.3. Quality Assessment Results of Expert Consensus Statements

Six expert consensus documents were included in this study [3,14,17,24,25,26]. Two documents were rated as “unclear” for item 6 (“Are there inconsistencies between the proposed views and previous literature?”), while the remaining four received “yes” ratings for all items. The expert consensus documents were generally of high quality with comprehensive development, and all were included in the final analysis (Table 3).

#### 3.2.4. Quality Assessment Results of Systematic Reviews

This study included one systematic review [27]. Except for items 9 (“Was potential publication bias assessed?”) and 10 (“Were recommendations for policy and/or practice supported by reported data?”), which were rated “No,” all other items were rated “Yes.” The overall quality assessment was high, and the review was included.

### 3.3. Evidence Synthesis

After extracting and synthesizing the evidence on skin lesion management in Pemphigus patients, 14 articles were included for final analysis. The best available evidence across five key areas was integrated: management principles, skin assessment, lesion care, health education, and recurrence with follow-up. This process yielded 24 evidence-based recommendations, as presented in Table 4.

## 4. Discussion

This evidence review provides a structured, evidence-based framework for managing Pemphigus-related skin lesions and wound care. The comprehensive standardized clinical protocol developed transcends traditional nursing principles, offering actionable guidance for clinical practice. Subsequent sections will elaborate on the core clinical implications throughout the entire care process.

### 4.1. Implementing a Multidisciplinary Collaborative Management Model to Strengthen Early Comprehensive Assessment

Evidence 1–3 indicates that Pemphigus patient management should follow the principles of multidisciplinary collaboration and early comprehensive assessment. As a chronic and recurrent autoimmune disease, effective Pemphigus management depends not only on accurate medical diagnosis and treatment but also on early and systematic nursing involvement. Bilgic et al. found that without timely intervention, Pemphigus mortality rates can reach 90% [28]. Nurses play a crucial role in monitoring skin lesion changes and assessing self-care capacity, enabling early detection of disease progression and providing key support for timely intervention. Furthermore, multiple studies confirm that a dermatologist-led multidisciplinary team can deliver more scientific and standardized management based on disease severity and treatment response [16,18,21,22]. Clinical practice demonstrates that a specialized nurse-led multidisciplinary model optimizes Pemphigus care: through early assessment and standardized wound care, nurses promote healing while coordinating with the team to adjust treatment plans, thereby collectively improving patient outcomes [29,30].

In summary, we recommend systematically implementing multidisciplinary collaborative management for Pemphigus patients, emphasizing the pivotal role of nurses in condition monitoring, nursing implementation, and holistic care to achieve effective skin lesion control and improved quality of life.

### 4.2. Systematic Skin Lesion Assessment Provides Objective Basis for Nursing Practice

Standardized assessment forms the foundation for clinical diagnosis and treatment, as well as skin lesion management. Evidence 4–5 recommends using validated skin assessment tools based on clinical presentation and test results to support objective evaluation and monitoring [15]. The Pemphigus Disease Area Index (PDAI) is internationally recognized as the core assessment instrument, with scores ranging from 0 to 263, where higher values indicate greater disease severity [31]. Compared to relying solely on subjective descriptions, effective assessment tools provide traceable, comparable data on skin lesion progression and treatment response. Additionally, the enzyme-linked immunosorbent assay (ELISA) is commonly used to assess disease activity by measuring anti-desmoglein antibody levels, providing laboratory data to complement clinical evaluation [16,18,21].

Antibody levels show close correlation with disease activity. Research indicates that persistently high anti-Dsg1 antibody levels often predict increased risk of skin lesion recurrence [22]. Thus, nurses should monitor patients for new blisters or expansion of existing lesions, promptly report these findings to the healthcare team, and maintain accurate records. Evidence shows that systematic assessment-based nursing practice enables more precise disease management and helps reduce related complications [28].

In summary, systematic and multidimensional nursing assessment using standardized tools such as PDAI and ELISA provides dynamic, measurable data on skin lesions and supports evidence-based nursing decisions, ultimately improving both the quality and safety of patient care.

### 4.3. Early Wound Protection and Comprehensive Care Accelerate Skin Lesion Healing

The refined techniques for wound care demonstrate distinct treatment approaches for different stages of skin lesions. Evidence 6–12 outlines key aspects of blister and skin lesion care in Pemphigus patients. Appropriate wound care is essential to prevent secondary infections and support healing. Care priorities differ depending on disease severity and treatment setting. For hospitalized patients with extensive lesions, severe conditions, or multiple comorbidities, nurses should focus on acute wound management and symptom control. For outpatients with limited lesions and milder disease, the emphasis should be on health education and treatment adherence [24].

For intact blisters, avoid pressure or friction; if blister tension is high, perform sterile needle drainage while preserving the blister roof, then apply antibiotic ointment to maintain dryness [15,16,22]. For ruptured or purulent lesions, clean with chlorhexidine and cover with functional dressings like paraffin gauze or emollients [16,18,21,22,25]. For itchy crusted lesions, gently wipe with sterile saline instead of removing scabs [14]. Oral care should include saline-based cleansing with cotton balls, soft-bristled toothbrushes, and antimicrobial mouthwash to reduce mucosal injury, infection risk, and pain [14,27].

Studies indicate that Pemphigus patients are prone to pressure injuries due to limited mobility and impaired circulation [22]. Those with severe lesions should be managed with pressure-relieving mattresses and regular position changes. Given the dynamic nature of Pemphigus with its potential for recurrence, care should include personalized wound management strategies, close monitoring for lesion changes and infection signs, and thorough documentation. This comprehensive approach optimizes both treatment and nursing care to improve patient recovery.

### 4.4. Early Identification of Complication Risks and Implementation of Comprehensive Health Education

Systematic and comprehensive health education can significantly enhance patients’ self-efficacy and treatment adherence, indirectly promoting improved clinical outcomes. Evidence 13–22 addresses complication prevention and daily management in PV patients, with strong evidence supporting clinical application. Immunosuppressed patients receiving oral corticosteroids (>20 mg/day prednisolone for ≥2 weeks) or immunosuppressive therapy should be vaccinated against influenza and pneumococcal infection [32]. These patients are particularly vulnerable to severe infections, including sepsis [33]. Early recognition, prompt antibiotic treatment, maintaining tissue perfusion, and managing organ dysfunction are crucial for improving outcomes [16,21,25]. The Sequential Organ Failure Assessment (SOFA) score should be evaluated every 48 h to monitor organ dysfunction in critically ill patients [21].

Studies indicate that Pemphigus patients have approximately 5% risk of venous thromboembolism (VTE) within the first year after diagnosis [16,18]. Although antithrombotic prophylaxis is essential for reducing VTE events, it may increase the risk of skin bleeding and erosion. Nurses should therefore assess both VTE risk and skin integrity, implement protective skin care during anticoagulation, and educate patients to monitor for redness, warmth, or pain. Multidisciplinary collaboration is crucial—nurses should communicate closely with physicians to adjust heparin dosage, consider compression dressings, and monitor patients every 4 weeks or more frequently to minimize adverse events [34].

To effectively control disease progression, a multidimensional daily management approach should be established that combines complication prevention and lifestyle interventions. This includes early identification and systematic monitoring to reduce complication risks, along with guiding patients to modify lifestyles to strengthen immunity and support skin repair [15]. While one international study suggested potential links between dietary components (thiols, isothiocyanates, phenols, tannins) and Pemphigus [35], this association requires further confirmation. Currently, patients are advised to maintain balanced nutrition, use sun protection outdoors, and avoid crowded places to minimize infection and injury risks [14].

Psychosocial support represents an essential component of comprehensive Pemphigus management. Disease severity frequently correlates with mental health deterioration, as visible skin lesions often cause significant emotional distress. Evidence indicates that anxiety treatment not only helps regulate mood but may also indirectly support lesion healing [36]. Health education should therefore improve recognition and screening of co-existing psychological issues while enhancing mental health support pathways [37]. Patients are encouraged to obtain information from reliable sources and join support groups for experience sharing and emotional management, which can alleviate anxiety and depression while improving quality of life [17,18,22].

Current health education for Pemphigus patients, while often adjusted for disease severity, lacks standardized guidance in key areas including complication recognition, home management, and systematic prevention. Existing research primarily focuses on basic medical treatments, with insufficient high-quality evidence regarding systematic health education’s role in improving self-management capabilities. Future studies should develop structured health education programs that are phased, comprehensive, and practical, with clearly defined outcome measures. These evidence-based protocols would help clinicians balance treatment benefits against risks, with the ultimate goal of enhancing long-term patient outcomes.

### 4.5. Long-Term Regular Follow-Up Is Key to Improving Patient Outcomes

Evidence 22–23 addresses Pemphigus recurrence monitoring and follow-up care. Clinical follow-up should assess disease severity, treatment response, and adverse effects, with frequency adjusted based on these evaluations [15]. Studies indicate that approximately half of patients experience recurrence after treatment completion, with some relapsing years after medication discontinuation [3]. When recurrence is confirmed, treatment regimens (including corticosteroids) should be promptly adjusted under medical supervision, with a comprehensive assessment focusing particularly on skin lesion changes [23].

For follow-up frequency, evidence recommends phased individualized scheduling: during active disease phases, follow up every 2–4 weeks until stabilization; every 4–8 weeks once controlled, continuing until corticosteroid discontinuation; then, switch to quarterly follow-ups after achieving complete remission with normalized anti-Dsg antibody levels [18]. Nurses should guide patients in maintaining skin lesion documentation and monitor for recurrence signs and wound status during follow-up visits [15,16,18,21,22].

In summary, standardized relapse management and structured long-term follow-up are essential for improving Pemphigus patient outcomes. In clinical practice, an individualized approach should guide follow-up frequency based on disease severity and patient needs. Regular assessments through this tailored schedule ensure effective use of medical resources and sustained treatment benefits.

### 4.6. Research Limitations and Future Directions

This evidence review synthesizes the literature from multiple regions, including guidelines and expert consensus documents from China. The management approaches for skin lesions emphasized in these sources reflect regional healthcare resources and clinical philosophies. Therefore, when adopting evidence, adjustments should be made according to local healthcare models. Additionally, due to the rarity of the disease, high-quality interventional evidence remains insufficient, constituting a major limitation of this study. Future research should prioritize filling critical gaps, including conducting rigorously designed prospective interventional trials to validate the efficacy of standardized skin lesion management protocols on wound healing, infection, and other symptoms. Comparative studies evaluating different wound dressings and care regimens are also essential. Such research will provide urgently needed clinical guidance, facilitating the translation of this evidence framework into more personalized and standardized clinical practice standards, ultimately achieving simultaneous improvements in dermatological lesion management and patient quality of life.

## 5. Conclusions

This study systematically reviewed and synthesized the best available evidence on skin lesion management in Pemphigus patients. The evidence covers five key areas—management principles, skin assessment, lesion care, health education, and recurrence with follow-up—and integrates 24 evidence recommendations. These findings provide a systematic and scientifically grounded basis for the clinical management of skin lesions. However, due to the rarity of the disease, current research focuses mainly on drug therapies, with limited attention to skin lesion care and holistic disease management, representing a study limitation. Future research should develop high-quality nursing intervention studies to promote evidence translation and standardized application in clinical settings, thereby strengthening support for improving Pemphigus care quality.

## Figures and Tables

**Table 1:** Characteristics of included studies (*n* = 14).

Included in the Literature	Publication Year (Year)	Evidence Type	Evidence Source	Literature Topic
Michael et al. [20]	2024	Clinical Decision Support	Up to Date	Treatment of Common Pemphigus and Leaf-Falling Pemphigus
De et al. [21]	2025	Guideline	PubMed	Pemphigus Monitoring, Treatment, and Comprehensive Management
Chinese Medical Doctor Association Dermatology Branch [15]	2024	Guideline	Yimaotong Guideline Network	Chinese Guidelines for the Diagnosis and Treatment of Pemphigus
Schmidt et al. [16]	2020	Guideline	Web of Science	Systemic Maintenance Therapy for Pemphigus Patients
Masayuki et al. [23]	2014	Guideline	Embase	Management of Pemphigus Treatment and Monitoring
Joly et al. [18]	2020	Guideline	Guideline Network	Management of Pemphigus
Harman et al. [22]	2017	Guideline	PubMed	Assessment, Prevention, and Management of Pemphigus
Murrell et al. [24]	2020	Expert Consensus	PubMed	Definite Diagnostic Methods and Management of Pemphigus
Porro et al. [3]	2019	Expert Consensus	Web of Science	Treatment Recommendations and Prognosis for Common Pemphigus
Chinese Medical Doctor Association Dermatology Branch [14]	2023	Expert Consensus	Chinese Medical Association Dermatology and Venereology Branch	Health Education Consensus for Pemphigus Patients
Chu CY et al. [17]	2023	Expert Consensus	CINAHL	Comprehensive Management of Pemphigus Patients
Chinese Association for International Exchange of Medical and Health Care, Dermatology Branch [25]	2020	Expert Consensus	Wanfang Database	Diagnosis, Treatment, and Recurrence Management of Pemphigus
Chinese Medical Doctor Association Dermatology Branch [26]	2025	Expert Consensus	Wanfang Database	Management Recommendations for Bullous Pemphigoid
Morais et al. [27]	2024	Systematic Review	PubMed	Oral Care for Pemphigus

**Table 2:** Quality evaluation results of included guidelines (*n* = 6).

Included in Guidelines	Standardized Percentage Score for Each Region (%)						Number of Domains ≥ 60%	Number of Domains ≥ 30%	Recommendation Level (Level)
	Scope and Purpose	Participants	Rigor of Development	Clarity of Guidelines	Applicability of Guidelines	Independence of Compilation			
Schmidt et al. [16]	88.88	80.55	71.13	88.88	47.90	100	5	6	B
Harman et al. [22]	75.00	66.66	79.38	88.88	54.16	75	5	6	B
Chinese Medical Doctor Association Dermatology Branch [15]	75.00	83.33	78.35	91.66	41.66	95.83	5	6	B
Masayuki et al. [23]	66.66	63.88	56.7	91.66	47.91	96.42	4	6	B
Joly et al. [18]	91.66	61.11	52.57	91.66	58.33	75	4	6	B
De et al. [21]	91.66	77.77	43.29	83.33	58.33	95.83	4	6	B

**Table 3:** Quality evaluation results of included expert consensus (*n* = 6).

Expert Consensus	①	②	③	④	⑤	⑥
Murrell et al. [24]	Yes	Yes	Yes	Yes	Yes	unclear
Porro et al. [3]	Yes	Yes	Yes	Yes	Yes	unclear
Chinese Medical Doctor Association Dermatology Branch [14]	Yes	Yes	Yes	Yes	Yes	Yes
Chu CY et al. [17]	Yes	Yes	Yes	Yes	Yes	Yes
Chinese Association for International Exchange of Medical and Health Care, Dermatology Branch [25]	Yes	Yes	Yes	Yes	Yes	Yes
Chinese Medical Doctor Association Dermatology Branch [26]	Yes	Yes	Yes	Yes	Yes	Yes

**Table 4:** Evidence summary of prevention and management of skin damage in patients with Pemphigus.

Evidence Type	Evidence Content	Evidence Level (Grade)
Management Principles	1. It is recommended to establish a multidisciplinary team led by dermatologists, with participation from psychologists, nutritionists, and specialized nurses in treatment and care. This team should provide scientifically standardized management based on the severity of the patient’s condition and any complications [3,22].	5
	2. Management principles for Pemphigus focus on controlling skin and mucosal lesions, reducing adverse reactions, promoting lesion healing, and improving quality of life [3,15,18,20,21,24].	2
	3. The management phases of Pemphigus comprise induction, maintenance, and remission. Disease control is achieved first, followed by gradual dose reduction to sustain remission. Nurses provide standardized care and continuously monitor the condition to minimize adverse effects [3,18,21,22].	5
Assessment of Skin Lesions	4. Effective assessment of skin lesions involves first conducting a comprehensive medical history, followed by selecting valuable evaluation criteria based on clinical manifestations such as the location, size, morphology, color, and presence of infection signs of the lesions [3,15].	5
	5. Assessment tools employing the Pemphigus Disease Area Index (PDAI), Autoimmune Bullous Skin Disease Intensity Score (ABSIS), and Enzyme-Linked Immunosorbent Assay (ELISA) serve as indicators for evaluating the severity and activity of skin lesions [15,18,20,21,22,23,24].	2
Skin Lesion Care	6. Pemphigus can cause skin ulceration and erosion. It is recommended that dermatology healthcare professionals manage and care for skin lesions to promote wound healing [22].	5
	7. For extensive skin damage, use antiseptics such as chlorhexidine for antimicrobial skin treatment. Perform the procedure gently to avoid friction [2,18,21,22,25].	5
	8. Select appropriate wound dressings based on lesion size and exudate volume. Small erosive areas may undergo daily debridement and dressing changes. For larger erosive areas, use non-adherent dressings, secure them properly, and change regularly to minimize pain and prevent secondary infection [26].	5
	9. If blisters rupture, avoid peeling off the epidermis. First, puncture the blister with a sterile needle to relieve pressure, then gently press with sterile gauze until fluid drains. Apply an antibacterial solution or cream as a wet dressing, keep the wound dry, and record the number and location of blisters daily [14,15,21,22].	5
	10. For oral lesions, perform oral care using saline-soaked cotton balls and monitor the condition of the oral mucosa [18,25,27].	5
	11. During wound care, promptly identify signs of infection at lesion sites and provide nutritional support and pain management based on lesion severity [14,18,20,21,22].	5
	12. For patients at risk of pressure ulcers, nurses are advised to implement position management using pressure-relieving mattresses during care [18].	5
Health Education Guidance	13. Patients are advised to use soft-bristled toothbrushes, mild toothpaste, and antibacterial mouthwash. Maintaining oral hygiene can alleviate pain and ulceration associated with oral lesions [18,25,27].	5
	14. Recommend oral corticosteroids or seasonal influenza and pneumococcal vaccination for patients; prohibit live vaccines [17,18,24].	4
	15. Nurses should emphasize the importance of regular ophthalmic evaluations, particularly for patients on long-term steroid therapy, to assess cataract risk [18,24].	4
	16. Recommend thromboprophylaxis for bedridden patients, monitor lower limb skin color and temperature, instruct patients on compression stocking use to prevent VTE [18,21,24].	5
	17. Educate patients to maintain a healthy mindset, follow a balanced diet, supplement with adequate calcium and vitamin D, avoid scratching the skin or vigorous scrubbing, and refrain from using irritants on affected areas [14,15].	4
	18. Inform patients and their families about the disease, prognosis, and potential adverse events related to care during treatment. Remind them to be aware of potential recurrence factors [14,16,17,18,22,24].	5
	19. Recommend that patients seek reliable sources of information and support groups to gain more disease knowledge and experience, promoting overall disease management [14,18,21,22,24].	5
	20. Recommend providing psychological support to patients while monitoring for potential depression [15,18,21,24].	5
	21. Inform patients that immunosuppressive therapy carries risks of liver/kidney disease, bone marrow suppression, and infection, requiring prompt intervention if symptoms arise [17,18,23].	5
	22. Inform patients that initial treatment achieves disease control when no new blisters appear within 2 weeks, 80% of existing rashes heal, or monthly new lesions do not exceed 3 and heal within 1 week. This allows maintenance of the dose reduction phase with monitoring to prevent recurrence and adverse reactions [15,16,17,21,22,24].	5
Recurrence and Follow-up	23. If a patient’s antibody titer increases, indicating potential disease recurrence or exacerbation, nurses should intensify observation of skin lesion changes and monitoring of systemic symptoms. If recurrence occurs, patients should be advised to adjust their treatment regimen as directed by their physician, accompanied by targeted nursing interventions [15,18,22].	4
	24. Long-term, regular follow-up is recommended for Pemphigus patients to monitor lesion healing and self-care adherence, thereby enhancing disease management [14,16,17,18,21,22,24,25].	5

## Data Availability

No new data were created or analyzed in this study.
